# HEALTH CARE PROVIDER ATTITUDES ABOUT INTEGRATING FAMILY CAREGIVERS INTO CLINICAL ENCOUNTERS

**DOI:** 10.1093/geroni/igz038.2278

**Published:** 2019-11-08

**Authors:** Joan M Griffin, Rachel Havyer, Karen Schaepe, Catherine Riffin, Lauren R Bangerter

**Affiliations:** 1 Mayo Clinic, Rochester, United States; 2 Mayo Clinic, Rochester, Minnesota, United States; 3 Weill Cornell Medicine, New York, New York, United States

## Abstract

The presence of family caregivers in clinical encounters is becoming more common with the aging of the US population and the continued shift of care responsibilities from health professionals in clinical settings to family caregivers at home. Patients accompanied to clinical encounters by caregivers are more likely to be older, sicker, and have lower health literacy. Research shows, however, that providers often do not initiate any caregiver participation and when they do, conversations center on relaying technical medical information rather than preferences and capacity to provide caregiving assistance. Little is known about provider perceptions of engaging caregivers in clinical encounters. Using data from 20 semi-structured interviews with physicians from primary and specialty care, we identified 3 inter-related themes about engaging caregivers in clinical encounters: 1) ambivalence about caregivers’ role in clinical encounters; 2) trepidation about posing questions directly to caregivers; and, 3) beliefs that systemic barriers exist that inhibit integration of caregivers. Providers, especially in primary care encounters, chiefly view caregivers as sources of supplemental information or for absorbing or reinforcing clinical instructions for care at home. Providers also voiced concerns about the ethics of assessing caregiver capacity to provide assistance to the patient without having clinical authority to treat or adequate resources to provide to caregivers. Finally, providers identified structural barriers, including time constraints, for integrating caregivers into the clinical care team. Findings provide insight into provider attitudes on the caregivers’ role, a perspective that is essential for understanding opportunities and challenges for implementing caregiver interventions in clinical settings.

